# Performance of Long‐Read Single‐Molecule Real‐Time Sequencing for SARS‐CoV‐2 Genotyping in Clinical Samples

**DOI:** 10.1002/jmv.70539

**Published:** 2025-08-02

**Authors:** Pauline Trémeaux, Justine Latour, Camille Vellas, Sofia Demmou, Noémie Ranger, Antonin Bal, Jacques Izopet

**Affiliations:** ^1^ Virology Laboratory Toulouse University Hospital Toulouse France; ^2^ Toulouse Institute for Infectious and Inflammatory Diseases (INFINITy) INSERM UMR 1391 – CNRS UMR 5051 – University of Toulouse III Toulouse France; ^3^ Virology Laboratory, Institute of Infectious Agents, Laboratory associated with the National Reference Centre for Respiratory Infection Viruses Hospices Civils de Lyon Lyon France

**Keywords:** long‐read sequencing, SARS‐CoV‐2 genotyping, SMRT sequencing

## Abstract

Due to the continuous genetic evolution of SARS‐CoV‐2, numerous variants have emerged and different whole genome sequencing techniques, necessary for accurate virus typing, have been developed. In this study, we evaluated the performance of PacBio single‐molecule real‐time (SMRT) sequencing for SARS‐CoV‐2 typing. Reproducibility was assessed on two internal quality controls, whose median reading depths were 1154X and 1059X. The overall sensitivity on 1646 clinical samples collected between January 2023 and June 2024 was 83.6% and was correlated to the viral load. By comparison, the overall sensitivity of short‐read illumina sequencing over the same period of time on 271 samples was 90.8%. Although less sensitive, SMRT sequencing was more efficient for the identification of the two lineages in a co‐infection case due to the amplification of long fragments. Comparing the results obtained by the two techniques, 10 out of 50 samples were identified with the same clade but not the exact same lineage at the time of analysis, because of the very frequent updates of the Pango taxonomy. Nevertheless, we obtained very similar fasta consensus sequences with a maximum difference of 4 nucleotides, showing that both methods provide accurate typing of SARS‐CoV‐2, useful for epidemiological or clinical studies.

## 1 Introduction

The Severe Acute Respiratory Syndrome Coronavirus 2 (SARS‐CoV‐2), responsible for the COVID‐19 pandemic, has a single‐strand positive RNA genome of 29.9 kb which has evolved genetically since its appearance in late 2019, resulting in a succession of variants [1, 2, 3]. This is partly the result of mistakes of the RNA‐dependent RNA‐polymerase, although limited by the proofreading activity of the exoribonuclease [4, 5]. Another genetic evolution mechanism of SARS‐CoV‐2 is recombination, in case of co‐infection with two different viral strains [6, 7, 8]. SARS‐CoV‐2 strains are defined by Nextstrain clades and Pango lineages, two co‐existing nomenclature systems [9, 10]. Recombinant viruses are designated with the lineages “X..” in the Pango taxonomy. The World Health Organization (WHO) has also defined Greek letters to designate several variants of concern that impact global public health. However, this nomenclature is less and less used since all circulating clades and lineages since mid‐2021 belong to the Omicron variant [3, 11].

Although most of SARS‐CoV‐2 genetic changes have no or little impact on the properties of the virus, some mutations can be associated with an increased transmissibility, escape from monoclonal neutralizing antibodies used in therapeutics or escape from the host immune response [5, 12, 13, 14]. Consequently, such as for influenza viruses, the design of vaccines against SARS‐CoV‐2 must adapt to the most recent viral strains in circulation [15]. The emergence and circulation of new strains are therefore closely monitored thanks to genomic surveillance politics in several countries including France, with global collaboration at the WHO level [16].

This monitoring requires efficient and easy‐to‐use laboratory protocols, using mostly next‐generation sequencing (NGS) platforms that enable whole genome sequencing, a prerequisite for accurate lineage identification. Several assays or protocols have been developed, either by industries or independent laboratories, using mostly Illumina or Oxford Nanopore Technologies (ONT) sequencing platforms. Previous studies have shown that Illumina sequencing provides a higher raw‐read accuracy and a better coverage while ONT sequencing offers shorter hands‐on time and runtime and allows long‐read sequencing that may be advantageous for recombination detection [17, 18, 19]. We recently developed a whole genome amplification and sequencing technique using the Sequel IIe platform of Pacific Biosciences (PacBio) [6, 20]. Comparison of single‐molecule real‐time (SMRT) sequencing with other NGS approaches in the field of medical virology are limited, and even more scarce for SARS‐CoV‐2 typing. Only one study recently compared capture amplification followed by SMRT sequencing with amplicon‐based approaches followed by Illumina or ONT sequencing on 92 samples [21]; no discrepancy was observed in lineage assignment.

This study aims to assess the performance of a long‐fragment amplification and SMRT sequencing technique on a large panel of clinical samples. Results obtained with long‐read SMRT sequencing and short‐read sequencing on an Illumina platform were also compared.

## 2 Materials and Methods

### 2.1 Patients and Samples

From January, 2023 to June, 2024, SARS‐CoV‐2 complete genome sequencing was performed on respiratory samples taken from patients of the Toulouse University Hospital to characterize clusters, investigate severe cases of hospitalized and/or immunocompromised patients, and monitor local epidemiology. Samples found positive with a cycle threshold (Ct) value under 28 were included. Once a week and depending on the prevalence of the virus, 20%–100% of the positive samples of the day with a Ct value lower than 28 were also transmitted to the National Reference Centre for Respiratory Infection Viruses for sequencing, according to the French surveillance policy of circulating SARS‐CoV‐2 strains.

### 2.2 SARS‐CoV‐2 Detection

SARS‐CoV‐2 was detected using the SARS‐CoV‐2/Flu A/B/RSV Assay on the Panther Fusion System (Hologic) following the manufacturer's instructions [22]. In brief, 500 µL of viral transport medium were transferred to a lysis tube containing 710 µL of buffer. The instrument used 360 µL of this mixture for nucleic acids extraction. This assay targets two sequences located on the ORF1ab gene of SARS‐CoV‐2.

### 2.3 SARS‐CoV‐2 Quantification Using ddPCR

Tenth‐fold serial dilutions of two SARS‐CoV‐2 strains were carried out to compare the Ct values obtained on the Panther Fusion instrument in routine to an absolute viral quantification using digital droplet PCR (ddPCR). For the latter, nucleic acid extraction was performed using the QIAamp Viral RNA Mini kit (QIAGEN) from 140 µL of sample. The ddPCR was performed using the One step RT ddPCR kit for Probes (BIORAD) on QX200 Droplet instruments (BIORAD).

### 2.4 Long‐Read SMRT Sequencing Using the Sequel IIe Platform

SARS‐CoV‐2 full‐length genomic sequences were obtained using the PacBio Sequel IIe instrument and an amplicon‐based approach, as previously described [6, 20]. Briefly, nucleic acids were extracted from 180 µL of viral transport medium using the MGIEasy Nucleic Acid Extraction kit on a MGISP‐960 system (Beijing Genome Institute), following the manufacturer's instructions. Viral RNA was reverse‐transcribed using the Superscript IV VILO enzyme (Life Technologies) and random hexamers. Two separate multiplex PCRs of 15 and 14 amplicons were performed afterwards using the Q5 Hot Start High Fidelity DNA polymerase (New England Biolabs) and M13‐tailed barcoded primers. The primers for these 29 overlapping amplicons of approximately 1.2 kb were based on the Midnight design [23] and were adapted to cope with SARS‐CoV‐2 genetic evolution. A second PCR using barcoded‐M13‐primers was then performed using the Kapa HiFi HotStart Ready Mix (Roche Diagnostics). For each sample, the two PCR products were mixed volume by volume and quantified to make a normalized pool before purification. Up to 250 samples of SARS‐CoV‐2 or other various viruses from clinical samples (Human Immunodeficiency Virus, Hepatitis E virus, Human Papillomavirus, Cytomegalovirus, Respiratory Syncytial Virus) were pooled for the library preparation, using the SMRTbell Express Template Prep 3.0 kit, and the sequencing on the Sequel IIe instrument.

The whole genome sequences were built from the PacBio reads using a custom‐made Snakemake pipeline. The HiFi reads were directly generated by the Sequel IIe sequencer (v.6.3.0, https://github.com/PacificBiosciences/ccs), then demultiplexed and filtered (minimum of 3 passes, Q20) with Lima (v.2.6.0, https://github.com/PacificBiosciences/barcoding). The resulting reads were mapped to the SARS‐CoV‐2 reference genome (Wuhan‐Hu‐1 isolate, GenBank accession number NC_045512.2) with Minimap2 (v2.17 [24]), analyzed by pbAA (v0.1.3, https://github.com/pacificbiosciences/pbAA) and CoSA (Coronavirus Sequence Analysis, v9.0.0, https://github.com/Magdoll/CoSA) to construct consensus sequences with a minimum depth of 10X. Potential co‐infections were identified by a home‐made script which constructs a hypothetical VCF file and thus a consensus sequence, where majority and minority variants are reversed (between minimum and maximum frequency thresholds).

Lineages and clades were attributed according to the most recent versions of Pangolin (v4.1.3 to v.4.3.1 [10]) and NextClade (v2.9.1 to v3.0.0 [9]) at the time of analysis.

### 2.5 Short‐Read Sequencing Using the Illumina Platform

Nucleic acids were extracted using the MGISP‐960 system, as described above. SARS‐CoV‐2 full‐length genomic sequences were amplified using the amplicon‐based COVIDSeq‐Test (Illumina) and the ARTIC V4 primers. Samples were sequenced with 100 base pair (bp) paired‐end reads using a NovaSeq 6000 Sequencing system instrument (Illumina), as previously described [25]. After quality trimming, reads longer than 30 bp were aligned to the SARS‐CoV‐2 genome MN908947 using Minimap2. Duplicate reads were removed, realigned using abra2 and read‐ends were clipped using samtools. Variants present at a minimum frequency of 5% were called using freebayes and bcftools (complete pipeline available at https://github.com/genepii/seqmet [25]).

### 2.6 Statistics Analyses

For concordance analyses between both sequencing techniques, consensus fasta of Illumina sequences were retrieved on GISAID and reanalyzed, as were SMRT sequences, with a common version of Pangolin (v4.3.1) and Nextclade (v3.9.1).

Cohen's kappa coefficient was used to measure the pairwise concordance between the two sequences obtained for each sample [26]. For all sequences, the Nextclade report listed the substitutions, deletions, insertions and missing positions, necessary to calculate differences between the pair of sequences. Nucleotide positions covered only by one of the two sequences were discarded from the comparison.

A maximum‐likelihood tree with ultrafast bootstrap (1000 replicates and a GTR+F+R3 evolution model) was constructed from the consensus sequences of both technologies by IQTREE (v2.0.3) [27], previously aligned with MAFFT (v7.505) [28]. Sequences were trimmed at the extremities to homogenize length between the two protocols.

## 3 Results

### 3.1 Analytical Performance of SMRT Sequencing

The repeatability of the technique was assessed by sequencing three times in the same run an internal quality control (IQC), in‐house made from a cell culture supernatant diluted in Minimum Essential Medium (MEM). We obtained identical results for strain identification (21I_B.1.617.2), coverage (95.8% of the genome), mutations profile on the S gene (E156del, F157del, T19R, G142D, R158G, A222V, L452R, T478K, D614G, P681R, D950N) and comparable results for mean reading depth (1258–1760X). Reproducibility was assessed by sequencing two IQC in each run, made from strains 20B_B.1.1.254 and 21I_B.1.617.2 (Delta variant). Correct identification of both the clades and lineages was obtained, with a median coverage of 95.8% over 2023. As for repeatability, this was explained by the absence of amplicon 5, due to a mismatch in a primer whose sequence was modified in June, 2022. This sequence adaptation to the new Omicron variants 22A_BA.1 and 22B_BA.5 led to a poorer match with the sequences of older strains used as IQC but improved the results of patient's samples. Over 67 runs, the mean reading depths were 1059X (interquartile range (IQR): 434–1615) and 1154X (IQR: 758–1635) for clade 20B and clade 21I IQCs, respectively.

**Figure 1:**
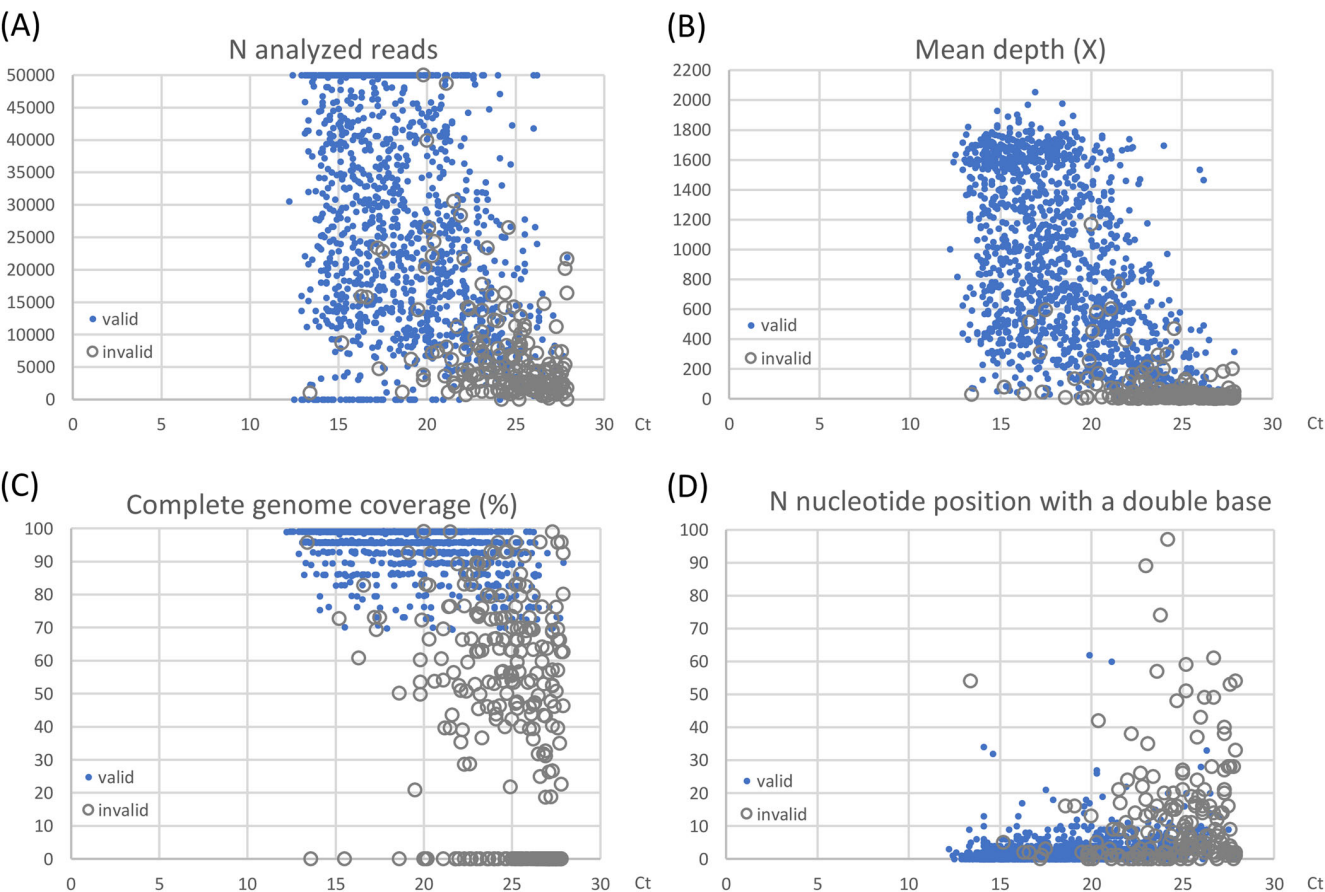
Performance characteristics of SARS‐CoV‐2 SMRT sequencing depending on the PCR Ct value. Ct values were obtained using the Panther Fusion system (Hologic). Blue dots represent valid results and gray circles represent samples that did not reach all validation criteria. In median, valid samples had 27464 reads analyzed (panel A), a reading depth of 832X (panel B), a coverage of 99.1% of the complete genome (panel C) and 1 nucleotide position with a double base (panel D).

During the 18‐months study period, a total of 1646 respiratory samples were sequenced on the Sequel IIe instrument: 1542 nasopharyngeal swabs, 38 expectorations, 26 tracheal aspirations, 33 bronchoalveolar lavage fluids and 7 saliva. The median Ct value was 18.9 (IQR): 16.1–22.3). In median, 24 039 reads per sample were analyzed (IQR: 10 607–44 812) (Figure 1A) for a mean reading depth of 700X (IQR: 263–1363) (Figure 1B). The median coverage was 95.8% of the complete viral genome (IQR: 89.3%–99.1%) (Figure 1C). Sequencing results with the SMRT technology were considered valid if the coverage was greater than 70% of SARS‐CoV‐2 genome including the key region of the receptor‐binding domain (RBD), or greater than 85% of the complete genome in case of an uncovered RBD region. Mutations in the RBD region can affect virus infectivity and/or be responsible for immune escape from antibodies. They are therefore often linked to the emergence and the designation of new SARS‐CoV‐2 variants [29, 30]. This explains the different coverage threshold for sequence validation. To detect and avoid potential cross‐contamination between samples, results were considered invalid if more than 10 nucleotide positions had a double base, unless these were confirmed during a second sequencing (Figure 1D). Such a high nucleotide diversity could be observed in cases of co‐infections (0.5%) or in immunocompromised patients (2.8%), some of whom developed chronic infections.

### 3.2 Sensitivity of SARS‐CoV‐2 Genotyping on Clinical Samples

**Figure 2:**
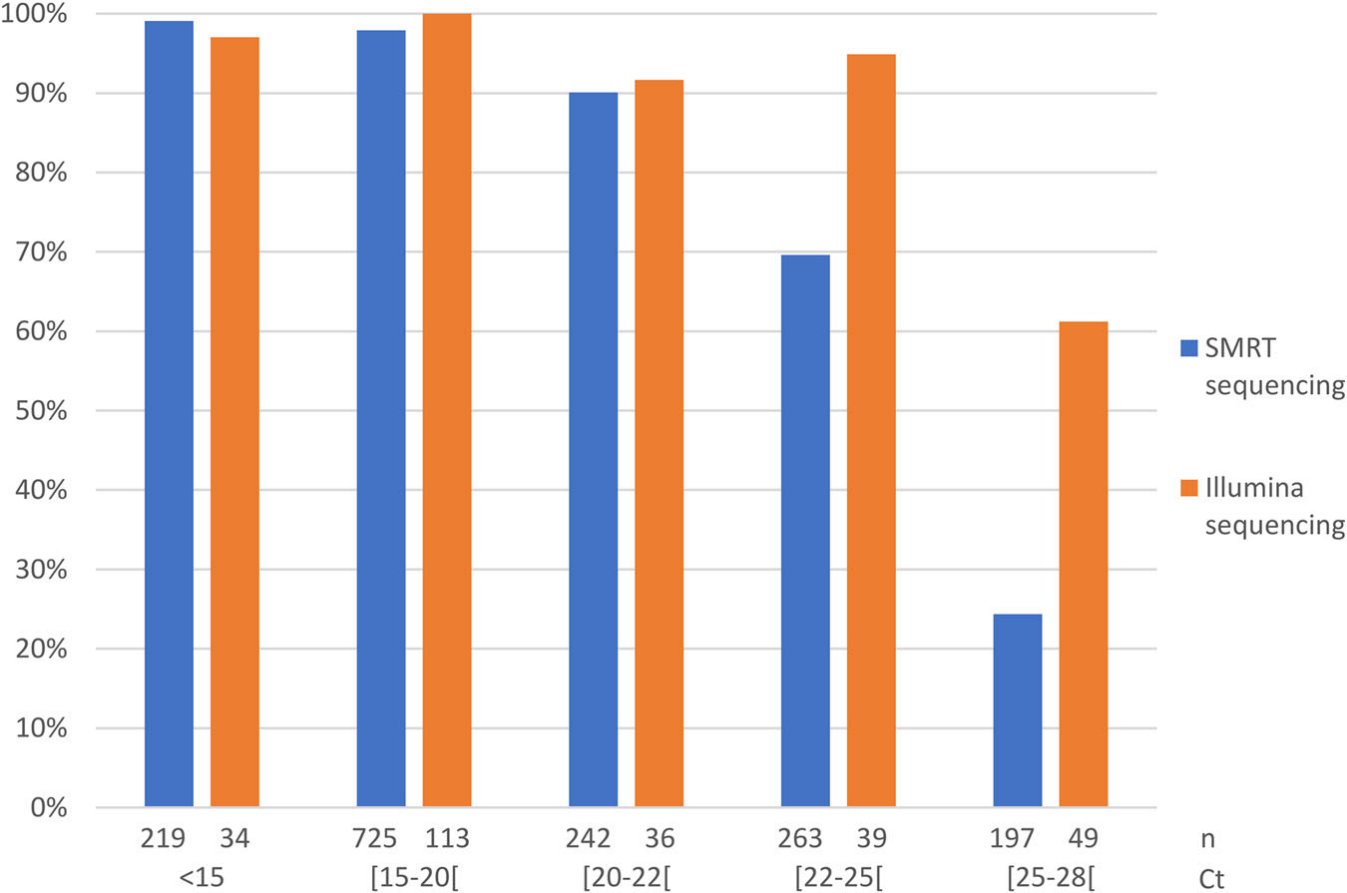
Sensitivity of SARS‐CoV‐2 whole genome sequencing on clinical samples. Sensitivities of SMRT and Illumina sequencing were compared according to the viral load estimated by the PCR Ct value.

The overall sensitivity of SMRT sequencing for clinical samples was 83.6% but varied depending on the viral load of the samples, and was therefore inversely correlated with the Ct value. The clade and lineage of SARS‐CoV‐2 were determined for 98% of samples with a Ct value under 20, 90% of samples with a Ct value between 20 and 22, 70% of samples with a Ct value between 22 and 25, and 24% of samples with a Ct value over 25 (Figure 2). Clades 23A, 23F, and 23I were the most frequent, in agreement with the prevalence of SARS‐CoV‐2 strains circulating in France at the time of the study (Supplementary document 1). To further precise the sensitivity of long‐read sequencing, we determined the absolute quantification of SARS‐CoV‐2 corresponding to these Ct values using ddPCR on two recent viral strains, 23F_EG.5.1 and 24C_KP.3. A Ct value of 20 corresponds to a viral load of 6.5–6.7 log cp/mL, a Ct value of 22 corresponds approximately to 6 log copies/mL and a Ct value of 25 corresponds to 5 log cp/mL (Supplementary document 2). To note, nasopharyngeal swabs accounted for 93.7% of samples but only 83.3% of amplification or sequencing failures. This might be explained by differences in viral loads. The global sequencing success rate was 85% for nasopharyngeal swabs (median Ct value: 18.7 [IQR: 16.0–22.1]), 70% for bronchoalveolar lavage fluids (median Ct value: 20.6 [IQR: 17.6–24.3]) and 50% for expectorations and tracheal aspirations (median Ct value: 21.8 [IQR: 18.5–25.2]).

### 3.3 Comparison Between Long‐Read and Short‐Read SARS‐CoV‐2 Sequencing

Over the study period, 271 samples were sent to the National Reference Center as part of national weekly epidemiological surveillance and sequenced with the short‐read Illumina sequencing platform. The median Ct value of these samples was 19.3 (IQR: 16.5–23.3), similar to those sequenced with the SMRT platform (*p* = 0.081). The clade and lineage of SARS‐CoV‐2 were determined for 99% of samples with a Ct value under 20, 93% of samples with a Ct value between 20 and 25, and 61% of samples with a Ct value over 25 (Figure 2). The overall sensitivity was 90.8%.

Among these 271 samples, 50 were sequenced in both laboratories (median Ct value of 19.1 [IQR: 16.7–21.9]). Three samples could not be amplified and sequenced by either of the two laboratories (Ct values superior to 26), while 3 samples had a result by only one of the two protocols (2 samples genotyped only by using Illumina short‐read sequencing and 1 sample only by using SMRT long‐read sequencing) (Table 1). The other 44 samples had concordant sequencing results. For 33 samples, identified clades and lineages were strictly identical and for 10 samples, clades were identical with both techniques while lineages were different (Table 1). To note, 5 samples among them were identified with Nextclade taxonomy as being recombinant viruses, but only one of the two laboratories identified concordantly an “X..” Pango lineage. The reanalysis of SMRT and Illumina consensus sequences with the same version of Pango gave identical lineage results for all samples except one (Sample 2: BQ.1.1 and BQ.1.1.23, respectively). Lastly, 1 sample was identified by both laboratories as being a probable co‐infection of two SARS‐CoV‐2 strains but only the long‐read SMRT sequencing protocol identified them (Sample 7; 23B_XBB.1.16.1 and 23A_XBB.1.5.28). This coinfection was confirmed by duplicate analyses with both sequencing techniques. To note, 12 samples had a different clade and/or lineage results at the reanalysis performed in December, 2024 compared to the initial typing result, because of the updates of Nextstrain and Pango taxonomies.

**Table 1:** Comparative SARS‐CoV‐2 typing results using SMRT and Illumina sequencing.

Sample Nb	Sample Date (dd/mm/yyyy)	Sample type	Ct Value	Initial SMRT sequencing result	Initial NRC Illumina sequencing result	Reanalysis results*	Remarks
1	09/01/2023	bronchoalveolar lavage fluid	27.5	*IND*	*IND*		
2	20/02/2023	nasoph. swab	27.3	22E_BQ.1.1	22E_BQ.1.1.23	Similar results as before	
3	20/03/2023	nasoph. swab	14.8	23A_XBB.1.5	23A_XBB.1.5		
4	27/03/2023	nasoph. swab	19.9	23A_XBB.1.5	23A_XBB.1.5.7	23A_**XBB.1.5.7** for both sequences	
5	02/05/2023	nasoph. swab	24.4	23A_XBB.1.5.28	23A_XBB.1.5.28		
6	22/05/2023	nasoph. swab	14.7	22F_XBB.1.9.2	22F_XBB.1.9.2	**23D_EG.4** for both sequences	
7	26/06/2023	nasoph. swab	14.1	co‐infection 23A_XBB.1.5.28 and 23B_XBB.1.16.1	co‐infection with 2 Omicron strains		
8	10/07/2023	nasoph. swab	18.6	23A_XBB.1.5.37	23A_XBB.1.5.37		
9	17/07/2023	nasoph. swab	16.5	22F_XBB.8.2	22F_XBB.8.2		
10	07/08/2023	nasoph. swab	15.1	22F_FE.1.1	22F_FE.1.1		
11	14/08/2023	nasoph. swab	22.0	23A_GF.1	23A_GF.1		
12	14/08/2023	nasoph. swab	21.7	23F_EG.5.1.3	*IND*		
13	14/08/2023	expectoration	16.7	23D_EG.1	23D_EG.1		
14	14/08/2023	nasoph. swab	20.5	23B_XBB.1.16	23B_XBB.1.16		
15	14/08/2023	nasoph. swab	23.2	23F_EG.5.1.3	23F_EG.5.1.3		
16	21/08/2023	nasoph. swab	14.5	23A_GK.2	23A_GK.2	**23G_GK.2.1** for both sequences	
17	28/08/2023	nasoph. swab	16.7	23F_EG.5.1.3	23F_EG.5.1.3		
18	11/09/2023	nasoph. swab	19.5	23F_EG.5.1.3	23F_EG.5.1.3	23F_**JG.1** for both sequences	JG.1 = EG.5.1.3.1
19	17/09/2023	nasoph. swab	16.8	23F_EG.5.1.3	23F_EG.5.1.3		
20	17/09/2023	nasoph. swab	16.8	23F_EG.5.1.3	23F_EG.5.1.3		
21	18/09/2023	nasoph. swab	16.7	23F_EG.5.1.1	23F_EG.5.1.1		
22	25/09/2023	nasoph. swab	19.1	23F_EG.5.1.3	23F_EG.5.1.3		
23	25/09/2023	nasoph. swab	21.0	23F_EG.5.1.1	23F_EG.5.1.1		
24	25/09/2023	nasoph. swab	17.2	23F_EG.5.1.1	23F_EG.5.1.1		
25	25/09/2023	nasoph. swab	17.1	23F_EG.5.1.1	23F_EG.5.1.1		
26	25/09/2023	nasoph. swab	23.0	23C_DV.7.1	23C_DV.7.1	23C_**DV.7.1.4** for both sequences	
27	25/09/2023	nasoph. swab	16.8	23F_EG.5.1.1	23F_EG.5.1.1		
28	23/10/2023	tracheal aspiration	26.4	*IND*	*IND*		
29	23/10/2023	nasoph. swab	15.1	23E_XBB.2.3.11	23E_GS.4.1	23E_**GS.4.1** for both sequences	GS.4.1 = XBB.2.3.11.4.1
30	23/10/2023	nasoph. swab	19.2	23F_HK.3	23F_HK.3	**23H**_HK.3	
31	23/10/2023	nasoph. swab	21.3	21L_BA.2.86.1	21L_BA.2.86.1	**23I**_BA.2.86.1 for both sequences	
32	23/10/2023	nasoph. swab	23.3	*IND*	23F_HK.3	**23H**_HK.3 (Illumina sequence)	
33	29/10/2023	nasoph. swab	22.1	23F_HK.3	23F_HK.3	**23H**_HK.3 for both sequences	
34	13/11/2023	nasoph. swab	20.1	23A_JD.1.1	23A_JD.1.1		
35	27/11/2023	nasoph. swab	14.2	23F_JG.3	23F_JG.3		
36	04/12/2023	nasoph. swab	16.2	23D_FL.1.5.1	23D_FL.1.5.1		
37	18/12/2023	nasoph. swab	20.7	23H_HK.3.1	23H_HK.3.1		
38	25/12/2023	nasoph. swab	14.9	23I_JN.1	23I_JN.1	**24A_**JN.1 for both sequences	
39	25/12/2023	nasoph. swab	22.3	recombinant_XDD	recombinant_ BA.2.86.1	recombinant_**XDD** for both sequences	XDD = EG.5.1.1/JN.1 recombinant strain; JN.1 = BA.2.86.1.1
40	21/01/2024	nasoph. swab	16.7	23I_JN.1	23I_JN.1.1	23I_**JN.1.1** for both sequences	
41	22/01/2024	expectoration	27.8	*IND*	23D_FL.1.5.1		
42	22/01/2024	nasoph. swab	16.6	23I_JN.1	23I_JN.1	**24A_JN.1.47** for both sequences	
43	29/01/2024	nasoph. swab	18.8	recombinant_JN.1	recombinant_XDK	recombinant_**XDK** for all sequences	XDK = XBB.1.16.11/JN.1.1.1 recombinant strain
44	29/01/2024	nasoph. swab	19.1	recombinant_JN.1	recombinant_XDK		
45	29/01/2024	nasoph. swab	17.6	recombinant_JN.1	recombinant_XDK		
46	12/02/2024	nasoph. swab	27.9	*IND*	*IND*		
47	10/03/2024	nasoph. swab	20.9	23I_JN.1	23I_JN.1	**24A_**JN.1 for both sequences	
48	25/03/2024	nasoph. swab	22.1	23I_JN.1	23I_JN.1	**24A_JN.1.45** for both sequences	
49	22/04/2024	nasoph. swab	14.6	24A_JN.1	24A_JN.1.16	24A**_JN.1.39.1** for both sequences	
50	29/04/2024	nasoph. swab	20.7	recombinant_XDD.1	recombinant_JN.1	recombinant_ **XDD.1** for both sequences	XDD = EG.5.1.1/JN.1 recombinant strain; XDD.1 = XDD + S:S740L

*Note:* Supplementary documents are available online only. Supplementary Document 1: Frequency of SARS‐CoV‐2 clades identified over 2023 and first half of 2024 in Toulouse, France. Supplementary Document 2: Absolute quantification of two SARS‐CoV‐2 strains using ddPCR. Supplementary Document 3. Detailed SARS‐CoV‐2 typing results using SMRT and Illumina sequencing. Supplementary Document 4. Phylogenic tree of SMRT and Illumina sequences.

Abbreviations: IND, indeterminate; nasoph., nasopharyngeal; NRC, National Reference Center.

* A reanalysis of consensus fasta sequences with Nextclade and Pango tools was performed in December 2024, while initial typing results were produced shortly after the sample date.

When looking at the fasta consensus sequences obtained by the two techniques, 40/44 (91%) samples had strictly identical sequences and a kappa concordance coefficient of 1. The other 4 samples had a kappa concordance coefficient between 0.982 and 0.996. The SMRT and Illumina sequences of 3/44 samples differed by only 1 single‐nucleotide polymorphism (SNP) while those of 1/43 sample differed by 4 SNPs. The latter case, with the most divergent consensus sequences, corresponded to the co‐infection (Sample 7) (Supplementary document 3). No difference was observed in the insertions and deletions in the consensus sequences obtained with the two techniques compared to the Wuhan reference sequence. Finally, phylogenetic analysis showed strong bootstrap values for sequences issued from the same sample with the two techniques, and they were clustered together on the phylogenetic tree (Supplementary document 4). Only sequences from Samples 23 and 24 were intermingled; they were taken on the same day from two residents of a long‐term care facility where there was a COVID‐19 cluster.

## 4 Discussion

Because of the intrinsic genetic variability of SARS‐CoV‐2, new variants have regularly emerged since the first apparition of the virus in late 2019. Monitoring of circulating viral strains is necessary for epidemiological studies and to adapt vaccine design to circulating strains. In clinical practice, virus lineage can guide the choice of antivirals or monoclonal antibodies used to prevent severe disease, and sequencing performed during follow‐up can detect the emergence of possible resistance mutations [5, 13, 15]. It requires efficient, robust and easy‐to‐use techniques. For SARS‐CoV‐2, precise typing is based on complete genome sequencing using NGS. During the COVID‐19 pandemic, several protocols have been developed and published. They were based either on capture enrichment or, more frequently, on PCR amplification with overlapping amplicons to have sufficient genetic material before the sequencing step [20, 31]. The challenge with the latter strategy is the genetic variability of the virus, which can lead to primers mismatches as the virus evolves and new variants emerge. Several updates to the design of the commonly used ARTIC primers have been published [32], while the Midnight panel has only been modified once after the emergence of the Omicron variants [23]. The reduced number of amplicons in the latter case limited the risk of mismatch with the viral genome and the need for primer modifications. Nevertheless, published updates are not always sufficient and localized drops in coverage can be observed [33]. A continued attention to the subject should therefore be implemented as part of the laboratories' quality approach. The use of an in‐house protocol might allow a higher flexibility of design and faster reactivity to virus evolution. Our technique is based on the Midnight design of 1.2kb‐long amplicons, but we made 10 primers changes since 2021.

The most widespread sequencing platforms were those using the short‐read Illumina technology or the Oxford Nanopore Technology allowing both short‐ and long‐read sequencing [18, 34, 35]. However, very few viral laboratories used the PacBio SMRT technology. One of its specificities is the possibility to sequence high‐length reads while preserving a high sequence quality [36, 37]. In this study, we showed on a large panel of samples that SMRT sequencing answered the needs of good sequence quality and high throughput for SARS‐CoV‐2 genotyping, confirming the data from our previous work [6, 20, 38]. Reproducibility was assessed by sequencing in‐house quality controls on each run with mean reading depths obtained over 1000X. We have also successfully used this protocol in our laboratory routine on several hundred clinical samples. Nevertheless, the amplification of long fragments limited the sensitivity of the technique. Indeed, the sensitivity decreased under 90% for samples over 22 Ct. Compared to nasopharyngeal swabs, SMRT sequencing was less sensitive for SARS‐CoV‐2 sequencing from other sample types, but this might be explained by lower viral loads. This should be confirmed on a larger number of samples of diverse types. In comparison, short‐read sequencing such as the Illumina technique offers a better sensitivity on low viral load samples: in our study, the drop of sensitivity was observed for samples over 25 Ct. On the other hand, long‐read sequencing can be advantageous for the identification of coinfections, as well as the study of quasispecies and minority variants using haplotyping [6, 39]. We have previously used this technique for instance for the follow‐up of immunocompromised patients with chronic infections, some of whom acquired key Spike mutations [40]. We have also reported a case of superinfection with a second SARS‐CoV‐2 clade that evolved to the selection of one predominant recombinant virus [6]. Long‐read sequencing also enabled the detection, in coinfected individuals, of several minority recombinant strains that could not have been identified only by variant calling alone, an analysis available for both short‐ and long‐read techniques [6]. Other teams have used this technology for diverse applications such as the study of HIV reservoir and quasispecies [41, 42], mixed human Cytomegalovirus strain infections [43, 44], the transmission bottleneck in Hepatitis B virus quasispecies [45] and for resistance genotyping of Hepatitis C virus [46].

In addition to sensitivity, we compared the consensus fasta sequences obtained with SMRT and Illumina techniques for the same samples. Although generated with different amplification, sequencing and bioinformatics methods, the sequences were highly similar (four nucleotide differences at most over the nearly 30 kb‐long genome). This attested to the performance and accuracy of the two techniques and the relevance of the validation criteria used. Both techniques can therefore be used in routine, depending on the equipment available in the laboratories. Despite identical sequences, lineages attributed to a same sample by the two laboratories sometimes differed. Those were not real discrepancies, as parental lineages and identical clades were identified in each case. This could be explained by the very rapid evolution of PANGO lineages, that required frequent updates of bioinformatic pipelines. Sublineages often vary from a single or a few mutations from their parental strains. A new lineage designation is also expected to represent one or more events of epidemiological significance, such as successive epidemic waves or movement of the virus into a new geographic area [47, 48]. However, this is not associated with clinical consequences in most cases. Although precise typing may be necessary for basic research and for epidemiological tracking of emerging new strains [49], use of Nextstrain clades might be sufficient in clinical practice.

## Author Contributions

Pauline Trémeaux and Jacques Izopet designed the project. Noémie Ranger, Justine Latour, and Sofia Demmou performed the PacBio sequencing and bioinformatics analyses. Antonin Bal was responsible for the Illumina sequencing analyses. Pauline Trémeaux, Justine Latour, and Camille Vellas analyzed the data. Pauline Trémeaux and Jacques Izopet wrote the initial draft. All authors revised and approved the manuscript.

## Conflicts of Interest

The authors declare no conflicts of interest.

## Supporting information

SMRT SARS‐CoV‐2 sequencing_supp doc 1–2.

SMRT SARS‐COV‐2 sequencing_supp doc 3.

SMRT SARS‐CoV‐2 sequencing_supp doc 4.

## Data Availability

The raw data are available upon request. Consensus sequences were published on GISAID (Virus name beginning with “hCoV‐19/France/OCC‐CHU‐TLS‐”; see Supplementary document 3 for accession numbers).
