# Supplementary material for: Performance of Long‐Read Single‐Molecule Real‐Time Sequencing for SARS‐CoV‐2 Genotyping in Clinical Samples
Source: J Med Virol. 2025 Aug 2;97(8):e70539. doi: 10.1002/jmv.70539 (PMC12317682; doi:10.1002/jmv.70539)
Supplement: Supplementary file 1 — SMRT SARS‐CoV‐2 sequencing_supp doc 1–2. [file JMV-97-e70539-s003.docx]

Supplementary Document 1. Frequency of SARS-CoV-2 clades identified over 2023 and first half of 2024 in Toulouse, France

SARS-CoV-2 clades were identified using full genome sequencing and the Nextstrain taxonomy (*Aksamentov I, Roemer C, Hodcroft EB, Neher RA. Nextclade: clade assignment, mutation calling and quality control for viral genomes. J Open Source Softw. 2021 Nov 30;6(67):3773*).

| Nexstrain clade | N samples | % samples |
| --- | --- | --- |
| 21K | 11 | 0.8% |
| 21L | 34 | 2.5% |
| 22A | 1 | 0.1% |
| 22B | 8 | 0.6% |
| 22C | 0 | 0.0% |
| 22D | 19 | 1.4% |
| 22E | 91 | 6.6% |
| 22F | 124 | 9.0% |
| 23A | 235 | 17.1% |
| 23B | 56 | 4.1% |
| 23C | 6 | 0.4% |
| 23D | 52 | 3.8% |
| 23E | 56 | 4.1% |
| 23F | 280 | 20.3% |
| 23G | 1 | 0.1% |
| 23H | 11 | 0.8% |
| 23I | 286 | 20.8% |
| 24A | 43 | 3.1% |
| 24B | 37 | 2.7% |
| 24C | 1 | 0.1% |
| recombinant | 17 | 1.2% |
| co-infection | 7 | 0.5% |

Supplementary Document 2. Absolute quantification of two SARS-CoV-2 strains using ddPCR


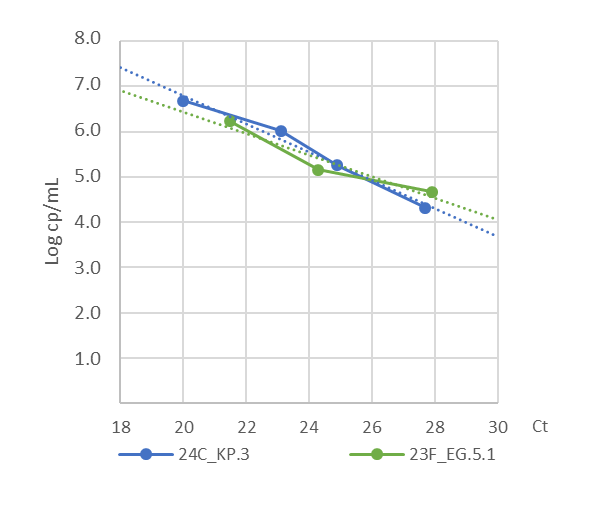


Quantification was performed on two of the most prevalent strains at the time of the study (January 2023 to June 2024). Ct values were obtained using the Panther Fusion system (Hologic). Linear regressions are shown in dotted lines.
