# Supplementary figures and images for: Performance of Long‐Read Single‐Molecule Real‐Time Sequencing for SARS‐CoV‐2 Genotyping in Clinical Samples
Source: J Med Virol. 2025 Aug 2;97(8):e70539. doi: 10.1002/jmv.70539 (PMC12317682; doi:10.1002/jmv.70539)

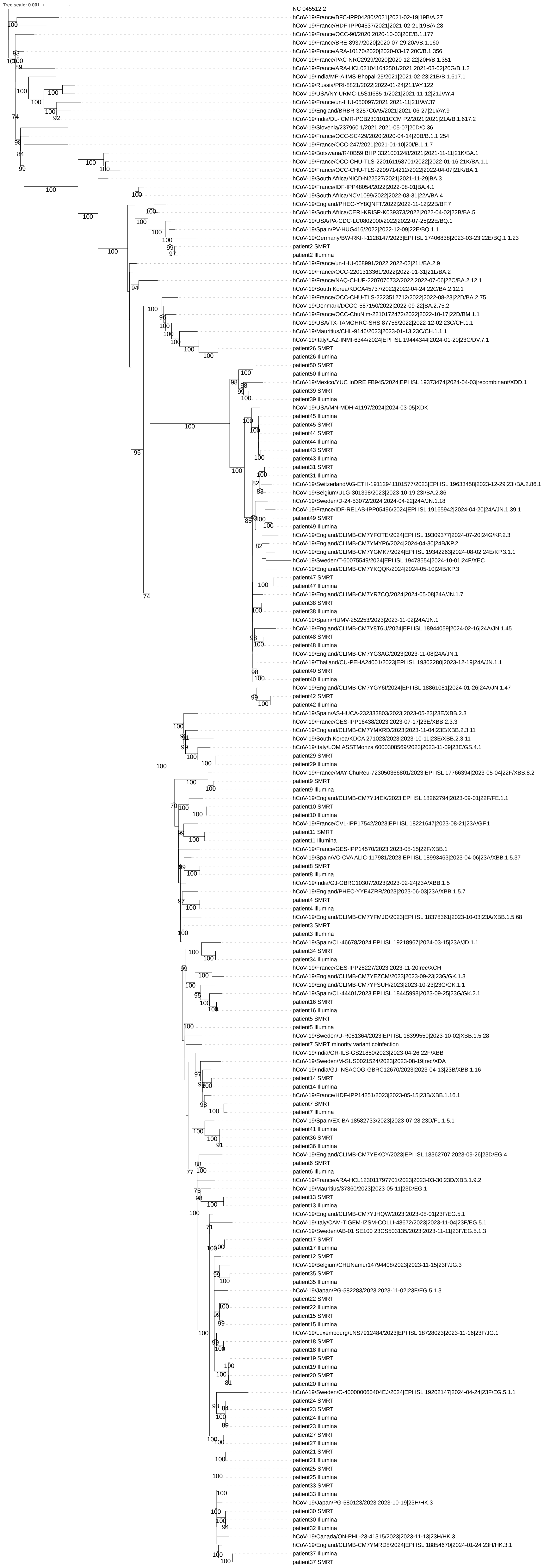

Supplement: Supplementary file 3 — SMRT SARS‐CoV‐2 sequencing_supp doc 4. [file JMV-97-e70539-s001.pdf]
